# Model for Managing the Integration of a Vehicle-to-Home Unit into an Intelligent Home Energy Management System

**DOI:** 10.3390/s22218142

**Published:** 2022-10-24

**Authors:** Ohoud Almughram, Sami Ben Slama, Bassam Zafar

**Affiliations:** 1 Faculty of Computing and Information Technology, King Abdulaziz University, Jeddah 21589, Saudi Arabia; 2 The Applied College, King Abdulaziz University, Jeddah 21589, Saudi Arabia; 3 Analysis and Processing of Electrical and Energy Systems Unit, Faculty of Sciences of Tunis El Manar, Tunis 2092, Tunisia

**Keywords:** demand side management, electric vehicle, home energy management system, home automation, multi-agent system, vehicle-to-home

## Abstract

Integration of vehicle-to-home (V2H) centralized photovoltaic (HCPV) systems is a requested and potentially fruitful research topic for both industry and academia. Renewable energy sources, such as wind turbines and solar photovoltaic panels, alleviate energy deficits. Furthermore, energy storage technologies, such as batteries, thermal, and electric vehicles, are indispensable. Consequently, in this article, we examine the impact of solar photovoltaic (SPV), microgrid (MG) storage, and an electric vehicle (EV) on maximum sun radiation hours. As a result, an HCPV scheduling algorithm is developed and applied to maximize energy sustainability in a smart home (SH). The suggested algorithm can manage energy demand between the MG and SPV systems, as well as the EV as a mobile storage system. The model is based on several limitations to meet households’ electrical needs during sunny and cloudy weather. A multi-agent system (MAS) is undertaken to ensure proper system operation and meet the power requirements of various devices. An experimental database for weather and appliances is deployed to evaluate and control energy consumption and production cost parameters. The obtained results illustrate the benefits of V2H technology as a prospective unit storage solution.

## 1. Introduction

### 1.1. Background

Humans have become more dependent on energy over time. Energy efficiency is necessary for intelligent household appliances and electric automobiles. Therefore, the conventional electrical grid confronts numerous challenges, including the inability to fulfil peak demand, expensive power outages, and pollution-induced environmental catastrophes. Because of these challenges and high energy consumption, power generating and distribution technologies must progress [1]. Clean and renewable energy helps prevent air pollution and increases the industry’s sustainability. Solar photovoltaic (SPV) or solar power systems can create solar energy without harming the environment. Consequently, solar energy produces less expensive energy than fossil fuels. Load balance and maximum power management both have downsides [2]. Accordingly, some smart grid (SG) technologies have evolved to solve these issues. In [3], the authors implemented smart metering to regulate the energy demand of smart homes (SHs). The latter is a crucial component of a SG to balance energy demand and generation. Additionally, it incorporates fog computing technologies for more precise load balancing. In [4], the authors conceptualized and constructed an SH prototype for storing SPV energy during power outages. The grid and SPV energy use were processed to compute costs, generate a bill, and identify the overall energy consumption. The load control technique has established several power consumption profiles for reducing peak demand. In [5], the authors published and designed sophisticated energy management for SHs. The proposed system integrated both wireless communication and renewable energy sources (RnEs).

In addition, the model combines a demand, supply, and peak load scheduling technique to reduce electricity bills and conserve energy. The authors created and developed an SH prototype in [6]. The suggested solution combined cloud infrastructure with IoT-enabled smart sockets to regulate their functioning and store device load patterns. In addition, an efficient real-time demand response (DR) scheduling system was created, reducing consumers’ electricity costs without altering their use habits. Load balancing and maximum power management are not the primary obstacles to power management. Energy management systems (EMSs) must be implemented to efficiently and locally manage each SMG. A building energy management system is an example of an environmental management system for businesses such as schools, hospitals, factories, etc. While a HEMS is intended primarily for homes and their appliances, home automation has been introduced as a potential innovation to provide uninterrupted electrical performance, resolve concerns with energy consumption, and enable intelligent devices to coordinate with new technology. Recently, energy businesses have worked to develop technologies that improve energy commercialization through energy system management and lower prices [7]. A HEMS is a potential form of home automation that can increase energy efficiency; it includes appliances, entertainment systems, security systems, environmental controls, etc. In [8], the authors proposed a HEMS strategy for efficiently scheduling energy resources in a SH. In addition, the suggested HEMS modeled the lifestyle-related functional dependencies of home appliances to assess the lifestyle requirements of the user. In [9], the authors described the self-scheduling of an efficient HEMS system using a stochastic framework of mixed integer linear programming. The suggested system determined the condition of household appliances throughout the day. In [10], the authors proposed an intelligent HEMS system based on a multi-agent system (MAS) to meet loading criteria and carry out the necessary system tasks. The results indicated that a smart HEMS system could intelligently plan the on/off status of a home by controlling the system’s conditions. This improved the SH’s energy efficiency. In fact, a HEMS design incorporating a RnE source and an energy storage system (ESS) was built and considered. Particle swarm optimization and binary particle swarm optimization have been utilized to enhance mathematical methods for general energy costs. According to the findings, the proposed structure saved up to 19.7% more money than prior efforts. Utilities have implemented time-of-use (TOU) pricing for residential customers. During times of heavy demand, this tariff increases the price of electricity. Maintaining a steady SG necessitates an efficient method for reducing the peak-to-average power demand ratio (PAR). In [11], the authors suggested an approach for home loads that incorporated SPV devices with battery storage. In this configuration, grid electricity was the primary source, with battery storage serving as a backup during moments of peak demand. Using ant colony optimization, the authors of [12] developed and presented an improved HEMS that reduced peak demand and improved user comfort. A comparison of the achieved results to non-scheduled alternatives demonstrated the expanded potential of HEMS to reduce PAR by scheduling household appliances depending on the terms of use pricing. In [13], the authors devised and discussed a HEMS strategy for SHs. The proposed strategy tried to facilitate peak-to-off-peak transitions while concurrently minimizing peak demand. In fact, it was built on a multi-objective scheduling issue that must be handled by addressing the objectives with a decision support problem (DSP), which helped maintain peak demand low and expenses low. An EMS system that employed an algorithm to automatically limit the power consumption of household appliances has been described. In [14], the authors stated that consumption was planned such that the highest load could be lowered for the least amount of money, based on user priorities. Prosumers and the creation of an effective HEMS system would benefit both end-users and system operators in terms of cost control and the reduction of peak-hour consumption costs. In [15], the authors presented a modular and automated HEMS system with three essential subsystems: load determination, prediction, and optimization. Energy consumption information was recorded and securely stored. The system was capable of adapting to each of the three subsystems. The most efficient subsystem substituted the least efficient subsystem in the background to maintain user comfort while ensuring the lowest cost of energy used. While in [16], the authors proposed a multipurpose HEMS system with variable rate tariffs in order to save costs and increase customer convenience. By controlling a household’s appropriate energy consumption pattern and consumer priorities and preferences, the HEMS could balance cost savings and comfort. In [17], the authors explored and suggested a prediction and optimization model for an intelligent HEMS system. The forecasting model took into consideration the uncertainty of future energy consumption and prices. In [18], the authors presented an optimization approach for scheduling household appliances. The results demonstrated that energy usage and utility bills were lowered as a result of the HEMS strategy. Previous research has suggested that an efficient HEMS system may overcome constraints such as peak demand, dynamic pricing, and consumption. Finding a thorough and optimal answer to various problems is my research area. DSM is an integral approach to protect operations and increase power reliability. A DSM plan addresses environmental concerns, high energy costs, limited power supply, and grid reliance issues. Some study have used DSM to improve the HEMS design. In [19], the authors studied how DSM can minimize energy usage and increase efficiency using new development methodologies. According to the research, SGs improve security and energy efficiency while retaining environmental awareness. In [20], the authors used a DSM with support vector regression (SVR) to estimate the number of days before distributed generation to tackle an intelligent home energy planning challenge. DSM considers equipment use, battery bank capacity, electricity price variations, and duty cycles. In [21], a MAS model based on DSM was proposed. SH agents managed how smart home gadgets were turned on and off. In [22], the authors presented a smart DSM and HEMS system. Several metrics were compared with and without the intelligent HEMS system to ensure the technology would operate, including maximum switchable power, maximum standby power loss, energy savings, and peak shaving capabilities. Electric vehicles (EVs) enhance fossil fuel independence, energy variability, and cost reduction in DSM. This SG component draws interest in modern power systems as a source of elastic demand. In [23], the authors presented some models and solutions to ensure the proper running of SG electric vehicles. A stochastic adaptive dynamic programming (ADP) approach to the HEMS model with SPV and EVs was provided. The models discovered HVAC charging and discharging schedule adjustment points for electric vehicles considering SOC, SPV generation, and outside temperature. Moreover, a predictive control framework was constructed to simulate a residential home with continual updates of the optimal hardware schedules. A comparison of the suggested model to the standard model indicated that it could cut energy expenditures while preserving thermal comfort and decreasing EV SOC problems. In [24], the authors examined customers’ scheduling preferences for electric cars, ESSs, and RnEs. In addition, ESS and EV charging and discharging technologies were integrated into a HEMS. This technology provided cost-effective solutions and enhances battery life. The proposed model’s MILP reduced costs and maintained user convenience by scheduling intelligent home energy. In [25], the authors developed a framework for charging EVs using the local energy model and HEMS system. Simulations of high EV adoption in a community indicated the framework’s efficiency in reducing peak demand and nighttime energy use. In [26], the authors described a centralized way to improve energy management for BIM components. The analysis considered SPV, EV, metering infrastructure, and loads for the BIM model. The BIM model could run thermal loads and could fulfil power demand flexibly. Managing the BIM process correctly enhanced the energy balance between production and loads in the MG of the new HEMS system.

MAS applications that forecast energy effects are essential research areas because they simplify understanding and modeling RnE energy demand. In [27], the authors discovered greater use of market models, distributed control, system recovery, and optimization in MAS applications in small networks. In [28], the authors reviewed the literature on MAS network power management. The authors mentioned that no other design compared to a MAS. In [29], the authors created an SPV modeling system for efficient power generation. The energy management system displayed demand- and attribute-based scenarios. In [30], the authors proposed an energy-efficient intelligent home prototype. The MAS simulated RT-EMS power management (RT-ES-EM). Each agent’s energy consumption and production prices determined source circumstances. In [31], the authors suggested a HEMS framework to minimize power expenditures and boost PV for self-consumption while balancing comfort and spending. A multi-agent P2P energy trading format was proposed and discussed in [32]. The proposed technique promoted cost-effective energy balance by facilitating domestic supply and demand.

### 1.2. Gaps and Contribution

Previous studies have demonstrate that EVs in SHs can cut grid electricity at peak times. RnEs, appliances, and EVs must be scheduled optimally. A HEMS assesses SG and innovative home conditions to schedule intelligent home devices, and to charge and discharge electric vehicles appropriately. V2H technology coordinates EV and HEMS tasks in intelligent homes [33]. In [34], the authors suggested V2H and DR software. Network power and capacity optimization enhanced resilience, self-healing, and operational costs. The results showed that the proposed model could meet off-grid demand. In [35], presented a HEMS strategy for residential on-peak self-power. During a network outage, PHEV electricity was used to power a home. In [36], the authors proposed an innovative approach for energy charging and discharging coupled with a HEMS to establish V2H economic efficiency. Electric car charging and discharge should be based on off-peak electricity pricing. Grid-to-vehicle (G2V) saves 11.6% more than V2H. (G2V). An energy-management unit was implemented and discussed in [37]. The study compared V2H and conventional efficiency. Range and SPV subsidies were considered. SPV and V2H improved the valley’s electricity and SPV usage and resulted in economic benefits. Home-distributed PV and V2H panels could meet the home load demand on sunny and overcast days without a grid. Valley SPV and V2H accepted wet-day restrictions. From the previous works, it is seen that the proposed HEMSs have considerable research gaps:

- The vast majority of EMS-optimized systems are incompatible with V2H and/or SPV systems.
- There is a lack of study analyzing the effects of meteorological conditions and driving behavior on SPV production and consumption.
- Additional research should be conducted on V2H services for SPV power transmission and peak shaving.
- The economic sustainability of V2H’s two-way operation is still up for debate.
- There is a need to develop an optimal HEMS system with or without V2H and/or SPV.
- V2H services should be examined for SPV power transmission and peak reduction.
- An upgraded HEMS should consider weather, driving behaviors, and SPV advantages.
- The HCPV design should be examined economically.
- An optimal schedule should be developed for home appliances to determine the on/off load states and charging and discharging times for EVs and V2H systems.

Here, we simulate four cost-optimization scenarios based on state transition diagrams to determine the most cost-effective SH solution.

The remainder of the paper is structured as follows: In Section 2, we introduce the MAS and intelligent agent concepts and the V2H-HCPV cost-benefit and V2H energy balance models designed to lower the net expenditures of families; in Section 3, the scenario-based MAS scheduling method is created and discussed for the HCPV model; in Section 4, the test performance and results are summarized; finally, we outline our conclusions.

## 2. Methodology

### 2.1. Multi-Agent System (MAS) and Intelligent Agent Concepts

An agent is seen as an entity (software or tool) that operates in a defined setting and can interact independently with improvements to the environments. An agent interacts with its environment through a physical representation or by recording diagnostic data in a database accessible to others. The concept of SG can be extended to existing software and hardware. An “agent” is considered to be in a SG if: an energy system is located within its ecosystem, it responds to system status such as voltage and current changes, it suggests a high level of autonomy (individuality).

Thus, it is necessary to distinguish between agents and multi-agent systems (MASs) and their usefulness in solving technological difficulties. To do this, the term “agent” must be expanded to include “intelligent agent” (IA). Depending on the task at hand, different internal architectures have been suggested so that the agents of the system can work on their own as follows:

- Reactive agents are reactive variables with little environmental understanding. The first use of the Brooks skeleton is significant. This structure’s class hierarchy has a specific behavior. Each layer or behavior in the system is independent, although high-level activity will remove low-level behavior. This type of anatomy highlights why it does not need to be examined but can be taken up quickly if necessary. Factors can not change or get rid of uncertainty, and it may be hard to tell where a factor will be in a given situation.
- Regarding deliberative agents, an explanatory model contains deliberative agents in which judgments are made correctly. Agents with this type of anatomy form strategies to achieve their goals, making them better suited to solving mysteries and responding to unexpected events. Several Belief–Desire–Intention (BDI) models have been introduced for building commercial agents, as well as many alternative models. In this model, the agent’s beliefs represent his feelings about the world. The agent’s motives are his desires. This is the agent’s goal, but they will conflict if the agent has many objectives. An agent’s plans are the actions he must take to achieve his goals.
- Regarding hybrid agents, during the procedure, most agents are hybrids of reaction and reflection, with examples intrinsic to Turing machines.
- A multi-agent system (MAS) is essentially a system with two or more IAs being implemented. Indeed, in a MAS, there is no clear goal for the system as a whole; instead, this is achieved by bringing together several independent agents, each with a distinct local objective. In contrast, a MAS has proven to be the most intelligent management unit regarding power distribution. The decentralized structure of MAS technology enables the division of complex problems into subproblems through simultaneous processing to achieve the end goal, thus, reducing the computational overhead of a single machine. Extensive research has shown, for example, that intelligent elements could be used to control the power grid sustainably and to isolate the part of the system that is not working.

### 2.2. HCPV Design

**Figure 1:**
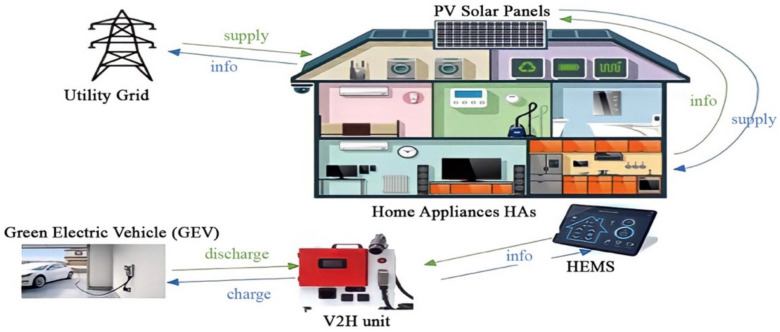
Smart home platform design.

**Figure A1:**
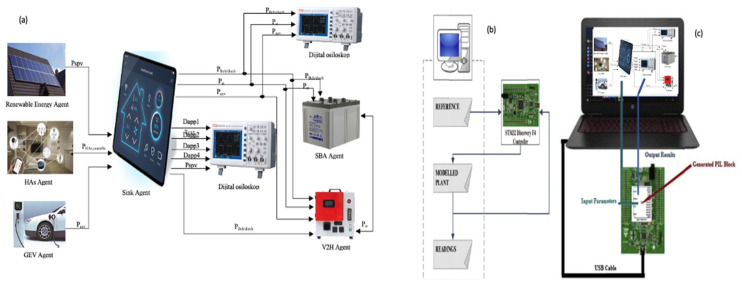
(**a**) Simulink prototype; (**b**) PIL simulation under STM32; (**c**) model generation.

As shown in Figure 1 and Figure A1, the HCPV design consists of a GEV, SPV, a HEMS, and HAs. The HCPV is designed to accommodate midday electricity demand. If the photovoltaic generation exceeds the load capacity, the excess power returns to the grid. A GEV battery generates electricity for a residence during peak hours. A bi-directional power converter, consisting of an AC-DC converter as a front end and a DC-DC converter as a backend, is used for the V2H unit. This bi-directional converter is useful for discharging the power from the GEV battery to the home. Thus, the GEV battery can be considered to be an energy source during peak demand. Furthermore, a stationary battery (SB) for storing excess energy is activated during a power shortage.

The following power system components will be designed for a smart home:

- Renewable energy sources (solar photovoltaic panels);
- Real-time intelligent energy management module (HEMS);
- HEMS storage device (batteries);
- Vehicle-to-home unit (V2H).

The agents in our proposed HEMS can make decisions, compare parameters, send real-time data, and send feedback about the system and consumption, which make the energy management process intelligent. Thus, the interactions among the subsystems become more efficient as compare with a HEMS without an MAS.

The energy balance equation for home automation using SPV or GEV stored energy is mainly represented in Equation (1). The following equations show that you cannot use both MG and GEV simultaneously and that the GEV must be constantly fed and released. Where the total SPV energy all day (P_spv,t_) is subtracted from the total power (m) produced from the GEV (P_GEV_) to obtain the grid power requirement (P_HCPV_). The relay state for the grid $\Psi_{1}$ should be specified either 0 or 1. Accordingly, P_HCPV_ at time t should be less than or equal to the maximum grid power P_grid-max_:

(1) $$\left\{\begin{array}{c} P_{\mathrm{HCPV}}\left(t,i\right)=\sum_{i=0}^{m-1}g\left(t,i\right)=\sum \sigma \left(k\right)P_{i\left(k\right)}-\;\sigma \left(0\right)P_{\mathrm{SPV},i}\\ =\overset{m-1}{\underset{i=0}{\sum }}\sigma \;\left(i\right)P_{\mathrm{GEV}}\left(t,i\right)+\sigma \left(0\right)\overset{m-1}{\underset{i=0}{\sum }}P_{\mathrm{GEV}}\left(t,i\right)-\sigma \left(0\right)P_{\mathrm{SPV},i}\\ =\sigma \left(i\right)g_{\mathrm{GEV},k}+g_{\mathrm{Home},k}-P_{\mathrm{SPV},t,s}\\ \sigma \left(0\right)=1,\;\forall t\\ P_{\mathrm{HCPV},i\;}\leq \Psi_{1}P_{\mathrm{grid}-\max}\\ \left\{\begin{array}{c} \Psi_{1}+\Psi_{2}\leq 1,\;\forall t\\ \Psi_{2}+\Psi_{3}\leq 1,\;\forall t \end{array}\right. \end{array}\right.$$

### 2.3. V2H-HCPV Cost Benefit Model

Depending on its configuration, a SH is equipped with a battery that provides power and energy storage or a GEV that powers and maintains the home. The method predicts the efficiency of the GEV throughout the charging and discharging schedule to reduce the user’s energy expenditure. In [22], the authors suggested an optimization process for determining the electricity cost for each period using the mathematical Equation (2) (The assumption is that the energy demand will remain constant $\gamma_{\tau }$ for the practical use of energy, C_net,min_ is the cost of household electricity (in USD), C_Has,SPE_ is the total cost of getting electricity (HAs, GEV, V2V) = 1, C_HCPV,SPV_ is the cost of installing a HCPV system, and C_HCPV,TR_ predicts total income for HCPV):

(2) $$C_{\mathrm{net},\min}={\;\gamma }_{\tau }\left({\;C}_{\mathrm{HAs},\mathrm{SPE}}+{\;C}_{\mathrm{HCPV},\mathrm{TR}}+{\;C}_{\mathrm{HCPV},\mathrm{SPV}}\right)$$

Rooftop solar P_SPVt,s_ is computed by combining solar P_SPVt,t_ at time t under typical rated conditions, SPV generated power, g_SPV,s_ solar irradiance s, and average temperature T_SPV,t_ rated under suitable conditions [38]. Where, P_SPV,t_ indicates the SPV radiation at time t, and $\emptyset_{\mathrm{SPV}}$ indicates the electricity temperature coefficient. It is written as Equation (3):

(3) $$P_{\mathrm{SPV},t,s}\left(\forall t\right)=g_{\mathrm{SPV}}^{\mathrm{rated}}\times \frac{g_{\mathrm{SPV},t}}{g_{\mathrm{SPV},s}}\left[\emptyset_{\mathrm{SPV}}\left(T_{\mathrm{SPV},t}-{\;T}_{\mathrm{SPV},t}\right)+1\right]$$

### 2.4. V2H Energy Balance Model

The energy stored at the end of time t is determined by Equation (4). In addition, the state of BT/V2H should reflect the amount of electricity remaining from the previous period (P_ST_) and the charge/discharge in the period t. Similarly, g(t) is the efficiency of charging activities ∆c, and discharging ∆d is evaluated to lower net household expenditures on energy costs [34]. As a result, P_ST_, the energy stored during time t, should be equal to P_ST_ (initial) which is the energy stored by the GEV at the beginning of period 1 (W/h). Here is a summary of the objective function:

(4) $$\left\{\begin{array}{c} P_{\mathrm{ST}}\left(t\right)=P_{\mathrm{ST}}\left(t-1\right)+P_{\mathrm{TR}}\left(t\right)+∆t\cdot g\left(t\right)\\ g\left(t\right)={∆}_{c}\times P_{c1}\left(t\right)-{∆}_{d}\times P_{c2}\left(t\right)\\ \forall t\;\in \left[1,\ldots T\right],∆t=1\\ t=1,P_{\mathrm{ST}}\left(t\right)=P_{\mathrm{ST}}\left(\mathrm{initial}\right) \end{array}\right.$$

### 2.5. Agent Renewable Energy System

**Figure 2:**
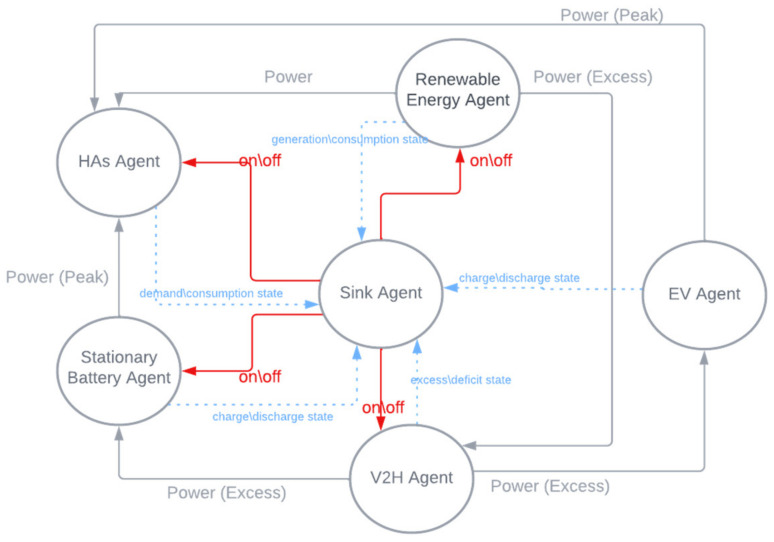
Agent interactions.

We demonstrated a hybrid system capable of managing energy demand among a MG, a SPV unit, and vehicle energy storage (GEV) in real time. The proposed system is designed to ensure that it is efficient and responsive to energy changes using an MAS. In practice, as shown in Figure 2, each system component has an independent unit that monitors its state and communicates with the primary agent.

▪ The primary agent (sink) coordinates with intermediate-level agents (renewable agent, EV agent, V2H agent, and HAs agent). Each IA can communicate with other implementing agencies to coordinate energy supply and demand through the basin. Additionally, the shared power state must be sent to all connected agents.
▪ The renewable energy agent collects real-time energy demand data to assess whether there is an energy surplus or deficit. The formula for estimating the rate of consumption for a particular purpose is given in Equation (5), where P_pv,comp_ indicates the SPV consumption rate in our situation; $P_{\mathrm{SPV},t}{\;\mathrm{and}\;P}_{\mathrm{GEV},t}$ are the power consumption of the SPV and the GEV, respectively, at time t; $P_{\mathrm{SPV},T}$ is the consumption of SPV at total time T:

(5) $$P_{\mathrm{SPV},\mathrm{comp}}=100\times \frac{P_{\mathrm{SPV},t}-P_{\mathrm{GEV},t}}{P_{\mathrm{SPV},T}}$$

where P_SPV,s_ is the amount of SPV energy consumed by the household load and P_SPV,t_ is the total amount of SPV energy used per day.

▪ The EV agent is responsible for charging and discharging the GEV batteries, which can be charged via SPV or the grid [34]. For the GEV network power transfer, the battery charger capacity is determined by $g_{\mathrm{GEV}}$, where P_GEV,t_ is the maximum power usage of a GEV when ψ2 is deactivated. When ψ3, the charging power (D_GEV,i_) must be less than the D_GEV_,_i,max_ limit (Equation (6)). Further, the effectiveness of the charged and discharged activities is taken into account.

(6) $$\left\{\begin{array}{c} g_{\mathrm{GEV},i}\leq \psi_{2},g_{\mathrm{GEV},i,\max}\\ D_{\mathrm{GEV},i}\leq \psi_{2},D_{\mathrm{GEV},i,\max} \end{array}\right.$$

▪ The V2H agent regulates the output power to provide backup power during power shortage conditions. The V2H unit involves a bi-directional charger to support the power converting function. The simulation considers a root mean square (RMS) 220 V for an AC supply and 350 V for the DC input at the front end. The battery is a hydrogen fuel cell 4.0 Ah capacity that contains 84 cells to produce from 0.5 to 0.08 V per cell.

The V2H agent is responsible for managing the nonlinear consumption of energy. The agent has complete control over the charging and discharging of the GEV battery. P_C,i_ represents the maximum power required to charge a GEV battery. The battery’s capacity must be equal to or less than the power of charge/discharge (P_Bch/disch_) The state of charge (SOC_GEV_) for GEV must meet certain conditions:

▪ The minimum SOC of the GEV should be less than or equal to the charging and discharging efficiencies (ηch,ηdis) of the maximum GEV’s SOC.
▪ The SOC should meet the daily driving distance (χ) to the maximum driving distance of the GEV (Dβ).

When SOC_V2H_,_i_ was introduced, it was shown that a charging converter (V2H) was present (Equations (7) and (8)):

(7) $$P_{\mathrm{ST}}\left(P_{\mathrm{Bch}/\mathrm{disch},i}\right)\;\leq P_{\mathrm{Bch}/\mathrm{disch},\max}\left(\mathrm{BT}/\mathrm{capacity}\right)\;$$

$$P_{ST}\left(t\right)\geq \;P_{\mathrm{GEV}-\min}\left(t\right)$$

(8) $$\left\{\begin{array}{c} {\mathrm{SOC}}_{\mathrm{GEV}\_\min}\leq {\mathrm{SOC}}_{\mathrm{GEVi}}\left(\eta ch,\eta dis\right)\times {\mathrm{SOC}}_{\mathrm{GEV}\_\max}\\ {\mathrm{SOC}}_{0}=100\%\left(1-\frac{\chi }{D\beta }\right)\\ {\mathrm{SOC}}_{V2H\_\min}\leq {\mathrm{SOC}}_{V2H_{i}}\left(\eta \mathrm{>c}\mathrm{>h},\eta \mathrm{>d}\mathrm{>i}\mathrm{>s}\right)\times {\mathrm{SOC}}_{V2H\_\max} \end{array}\right.$$

▪ Stationary battery agent (SBA) is a lead-acid battery with 56 Ah capacity and 12 V voltage. It is capable of storing about 0.55 kWh of energy. Thus, this SB is responsible for storing excess energy and the SBA is activated when a power deficit occurs. Equation (4) defines the stored energy at the end of time t. In addition, the condition of the BT/V2H should reflect the amount of electricity remaining from the previous period and the charge/discharge in the period t. In addition, the efficiency of charge/discharge processes are considered [35], where ∆t is the period of the elementary level; P_TR_ (t) is the amount of energy expended during trip at time t; P_c1_, P_c2_ are charged and discharged in time t (W/h).
▪ The HA agent is a physical presence agent that monitors the total household energy demand determined through the use of appliances (shiftable and non-shiftable), as defined in Equation (9) [36], where P_HAS_ represents the energy rating and α_HAs_ represents the state of the HAs, then the daily electricity consumption P_HAS,comp_ is expressed as:

(9) $$P_{\mathrm{HAs},\mathrm{comp}}=\overset{24}{\underset{t=1}{\sum }}P_{\mathrm{HAs}}\times {\;\alpha }_{\mathrm{HAs}\left(t\right)}$$

where α is the status of HAs which is represented as:

$$\alpha_{\mathrm{HAs}\left(t\right)}=\left\{\begin{array}{c} 0\;\mathrm{if}\;\mathrm{appliance}\;\mathrm{is}\;\mathrm{OFF}\\ 1\;\mathrm{if}\;\mathrm{appliance}\;\mathrm{is}\;\mathrm{ON} \end{array}\right.$$

Indeed, a MAS is a self-contained model consisting of many variables that cooperate to perform a complex task. Agents are used to accurately model consumption and production in response to changes in demand. Using a MAS, energy demand, system modeling, and simulation will be justified in order to coordinate the growth of energy storage in a way that benefits everyone. Table 1 summarizes the agents of the proposed system.

## 3. HCPV Scheduling Algorithm

### 3.1. HAs Scheduling Controller Condition

The majority of SH devices must be effectively classified and managed to lower the total cost of electricity, give convenience to customers, and reduce communication time. Home appliances are categorized based on their impact on user convenience and configuration according to user preferences and operational scheduling constraints [36]. Intelligent devices are shiftable and non-shiftable. The schedules for dishwashers and televisions are different. Non-shiftable appliances, such as a refrigerator, are required to operate at specific times. The console schedules shiftable appliances, such as a dishwasher, to run when electricity is less expensive. The hurdles are user convenience, electrical cost, and the peak-to-average ratio [37]. The objective function is defined to achieve this goal. While operating off-grid, the proposed HCPV can give information and electricity via the smart home, BT/V2H, and power. This connection may increase peak traffic on the leading network. Applications outside the network can handle this problem. A smart home is associated with both shiftable and non-shiftable everyday gadgets.

### 3.2. Scenario-Based MAS Scheduling Algorithm

**Figure 3:**
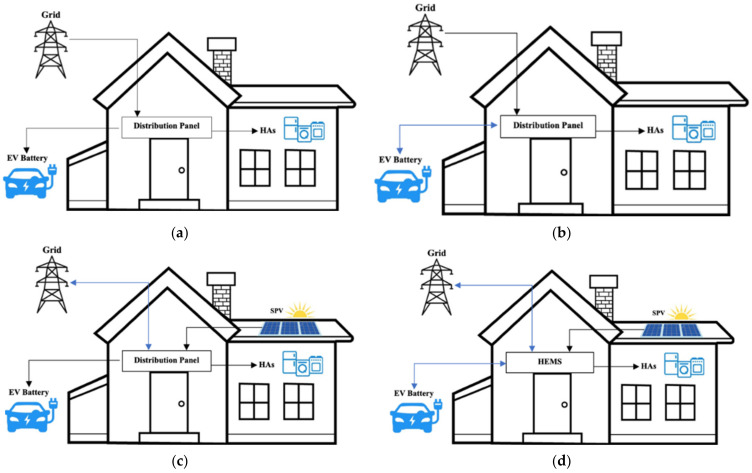
Energy flow in four different cases at a smart house. (**a**) State 1: standard GEV charging. (**b**) State 2: standard V2H charging/discharging. (**c**) State 3: GEV charging supplemented by SPV. (**d**) State 4: V2H charging/discharging supplemented by SPV.

This section illustrates alternative power distribution states to highlight the significance of incorporating V2H as an alternative energy source inside the HCPV framework. Figure 3 depicts the charging and discharging models of V2H and GEV utilized in a smart home with or without an SPV power supply. Standard GEV charging, GEV charging supplemented by SPV, standard V2H charging and discharging, and normal V2H charging and discharging supplemented by SPV are the power distribution states in this context. These states stipulate additional overall expenditures and energy balance standards, which necessitate appropriate scheduling patterns depending on specific scenarios. Our proposed framework tries to comprehend the impact of HCPV and V2H on power use efficiency while reducing expenditures. Included is real-time monitoring of the GEV’s produced and consumed power and power exchange among the microgrid, H2V, GEV, and SPV. A GEV form and a V2H form are submitted at a standard home.

#### 3.2.1. State 1: Standard GEV Charging

This state is devoid of SPV sources. In the conventional GEV charging state, both the intelligent home and GEV charging are powered by the utility grid. Consequently, the sink and EV agents collaborate with the HAs agent to regulate the supply and schedule, the net cost of utilizing electricity (C_net,min_). Equation (10) forecasts the average charging model for State 1, given that C_HCPV-TR_ = 0.

(10) $$\left\{\begin{array}{c} C_{\mathrm{HAs},\mathrm{SPE}}=C_{\mathrm{HAs}}^{1}+C_{\mathrm{HAs},p}\\ =\overset{T}{\underset{j=1}{\sum }}\mathrm{Pr}\left(j\right)\times P_{\mathrm{Ei}}\left(j\right)+{\Delta \mathrm{SOC}}_{\mathrm{GEV}}\times w_{\mathrm{GEV}}^{c}\\ \Delta {\mathrm{SOC}}_{\mathrm{GEV}}={\mathrm{SOC}}_{\mathrm{GEV}\;}\max-{\mathrm{SOC}}_{\mathrm{GEV}}\;\mathrm{peak}\\ \mathrm{where}\;w_{\mathrm{GEV}}^{c}=\frac{C_{\mathrm{GEV}}^{\mathrm{rated}}}{\eta \mathrm{ch}}\times P_{\mathrm{Ei}} \end{array}\right.$$

The variables Pr, P_EL_, $w_{\mathrm{GEV}}^{c}$, and SOC represent the HAs power load at time j, the electricity price at time j, the GEV state of charge, and the electricity price over 24 h, respectively. Moreover, the interaction between GEV and the grid ensures proper energy flow distribution (ΩSPV = 0), as seen in Figure 3a. While the SOC is integrated, grid electricity will charge the V2H/BT. To prevent the charger/converter from sustaining damage, the C_GEV_,_max_ measurement in Equations (6) and (7) regulate the power flow. If SOC is less than the maximum SOC_GEV_, the leftover power can charge the GEV/battery. Ultimately, excess electricity is provided by the grid.

#### 3.2.2. State 2: Standard V2H Charging/Discharging

During normal conditions, V2H supplies the energy to HAs without the inclusion of SPV, while the energy exchanged is sourced only from the grid. Therefore, the sink and V2H agents work with the HAs agents to manage the supply and timetable. In this state, the GEV supplies power to the HAs with a minimum state of charge (SOC_(V2H),min_) such that the total spending C_HAs,SPE_ = $C_{V2H}^{1}$. Consequently, Equation (11) is utilized to determine the total cost of $C_{V2H}^{1}$:

(11) $$\left\{\begin{array}{c} C_{V2H}^{1}=C_{V2H,p}+C_{V2H,\mathrm{GEV}}\\ =\overset{T}{\underset{j=1}{\sum }}\mathrm{Pr}\left(j\right)\times P_{\mathrm{Ei}}\left(j\right)+{\Delta \mathrm{SOC}}_{\mathrm{GEV}}\times w_{\mathrm{GEV}}^{c}\\ \Delta {\mathrm{SOC}}_{\mathrm{GEV}}={\mathrm{SOC}}_{\mathrm{GEV}\;}\max-{\mathrm{SOC}}_{\mathrm{GEV}}\;\min\\ \mathrm{where},\;w_{\mathrm{GEV}}^{c}=\frac{C_{\mathrm{GEV}}^{\mathrm{rated}}}{\eta \mathrm{ch}}\times P_{\mathrm{Ei}} \end{array}\right.$$

The spending $C_{\mathrm{net}}^{2}$ SPV and the grid produce the HA loads during particular time intervals (T_SPV_, T_GEV_). T_SPV_ is the usual period, and T_GPP_ is the nighttime peak period. In Equation (12), ∑Ωi is the installation cost (kW) (Ω = on (1), Ω = off (0), I ε [0 or 1]); indicates the net capacity, and g (δ) is the investment interest rate (1/Year (SPV lifespan)).

$$C_{\mathrm{net}}^{2}=C_{\mathrm{HOCP},\mathrm{SPV}}+C_{\mathrm{HAs},\mathrm{SPE}}^{2}+C_{\mathrm{HOCP},\mathrm{TR}}^{2}$$

(12) $$C_{\mathrm{HOCP},\mathrm{SPV}}=\sum \psi_{\iota }\times C_{\mathrm{HOCP},\mathrm{SPV}}^{2}\times \;g\left(\delta \right)$$

#### 3.2.3. State 3: GEV Charging Supplemented by SPV

SPV and GEV provide power to the HAs in this state, whereas only grid power is used for power exchange (see Figure 3c). SPV can power the HAs, whereas GEV is mostly a load (V2H = 0 and GEV = 0). Similarly, the sink, EV, and renewable energy agents collaborate with the HAs agent to manage the supply and schedule. Therefore, Equation (13) forecasts the total cost as CHAs, SPE ($C_{V2H}^{3}$)where Cnet, min = $C_{\mathrm{net}}^{3}$:

(13) $$\left\{\begin{array}{c} C_{\mathrm{HAs},\mathrm{SPE}}=C_{V2H,p}^{3}+C_{\mathrm{GEV}}^{3}\\ =\overset{T}{\underset{j=1}{\sum }}\mathrm{Pr}\left(j\right)P_{\mathrm{Ei}}\left(j\right)+{\;\Delta \mathrm{SOC}}_{\mathrm{GEV}}\times w_{\mathrm{GEV}}^{c}\\ {\Delta \mathrm{SOC}}_{\mathrm{GEV}}^{3}={\mathrm{SOC}}_{\mathrm{GEV}\;}\max-{\mathrm{SOC}}_{\mathrm{GEV}}\;\mathrm{peak}\\ \mathrm{where},\;w_{\mathrm{GEV}}^{c}=\frac{C_{\mathrm{GEV}}^{\mathrm{rated}}}{\eta \mathrm{ch}}\times P_{\mathrm{Ei}} \end{array}\right.$$

#### 3.2.4. State 4: V2H Charging/Discharging Supplemented by SPV

As indicated in Figure 3d, in this scenario, SPV, V2H/GEV, and the grid provide electricity to the HAs. As a result, the sink, renewable energy, V2H, and EV agents collaborate with the HAs agent to regulate the supply and schedule. Consequently, C_net,min_ = $C_{\mathrm{net}}^{4}$ and Equation (14) are used to anticipate the entire expense of ($C_{\mathrm{HAs},\mathrm{SPE}}^{4}$). Furthermore, the total revenue of C_HCPV-V2H_ = $C_{\mathrm{HCPV}-V2H}^{4}$:

(14) $$\left\{\begin{array}{c} C_{\mathrm{HAs},\mathrm{SPE}}^{4}=C_{V2H,p}^{4}+C_{\mathrm{GEV},V2H}^{4}\\ C_{\mathrm{HCPV}}^{4}=\overset{T}{\underset{j=1}{\sum }}\min\left(P_{\mathrm{spv}}\left(j\right)\right)\times \eta_{\mathrm{dis}} \end{array}\right.$$

**Figure 4:**
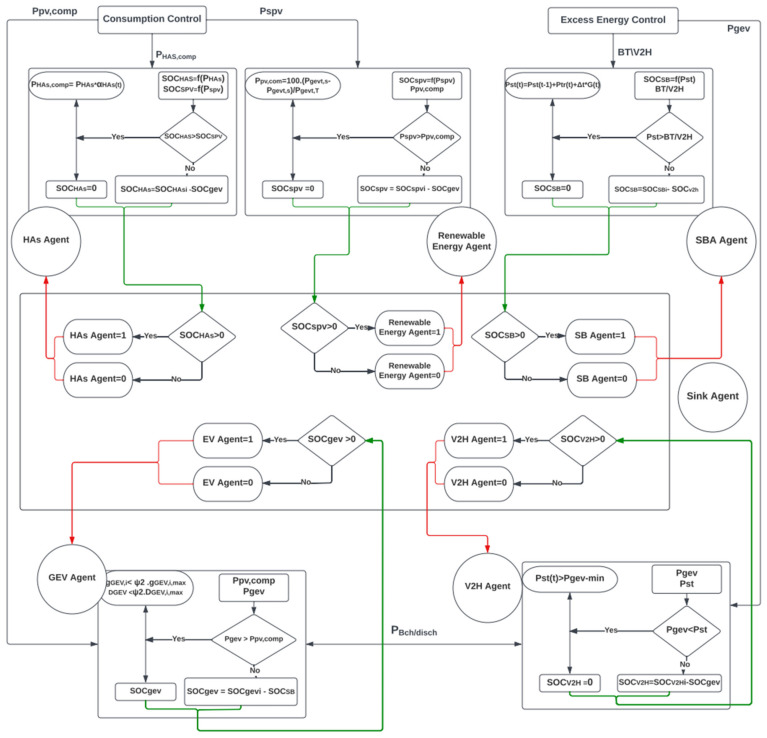
HCPV scheduling algorithm.

As compared with State 1, States 3 and 4 permit SPV to charge GEVs, which is more environmentally beneficial. However, merging V2H with HCPV mitigates economic obstacles, decreases emissions, and conserves energy. There is no difference in travel time or energy consumption across the scenarios. Additionally, V2H/GEV offers energy for the house load in addition to SPV and the grid. It can store the extra SPV energy in an electrical battery in the home’s power supply. This helps the user optimize their revenue. Figure 4 depicts the entire system behavior of agents’ communication and coordination, used to make the most suitable decisions. Each agent checks the energy consumption of each SG component. The state of each element is then communicated to the supervisor (sink) agent to coordinate the conditions of other agents. On the one hand, if a shortage in SPV power (P_spv_) is detected, the GEV agent will be activated to supply the power requirement. On the other hand, the SBA agent will be contacted if there is an excess of power.

## 4. Results and Discussion

**Figure 5:**
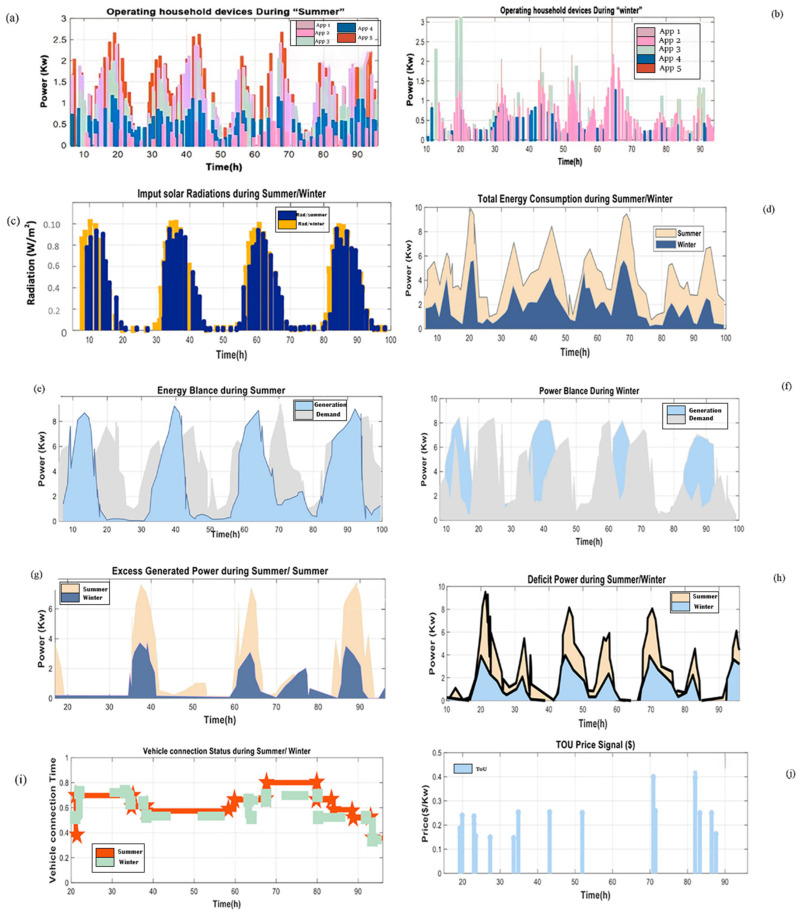
Input experimental profiles during “winter” and “summer”: (**a**,**b**) Operating household devices; (**c**) input solar radiation; (**d**) total energy consumption; (**e**,**f**) power balance; (**g**) excess generated power; (**h**) deficit generated power; (**i**) vehicle connection status; (**j**) TOU price.

In this study, electrical appliances, solar, GEV, and storage systems were all considered. The experimental simulation extracted accurate solar radiation and outdoor temperature profiles over four days from a Saudi meteorological database [38] (see Table A1). The consumption characteristics of major household appliances are depicted in Figure 5d. Indeed, this section contains simulation results that demonstrate the performance and efficiency of the proposed algorithm in reducing the daily electricity cost of a smart home. This study completed its calculations in less than 96 h. Bright sunlight and temperature data were derived from an experimental database. For simplicity, daily solar meteorological data were used to calculate the projected solar output curve during the summer and winter, as shown in Figure 5c,which shows that input irradiance is about 0.8–0.10 W/m^2^ during the period from 8 a.m. to 3 p.m. Figure 5d represents the total consumption, which reaches 10 kW during summer and 8 kW during winter.

Our study also found that household electricity use is higher in the summer months based on near-real-time electricity consumption data. In comparison, during winter, the electrical load by hour varies less, but peaks in the morning and evening. As a result, household energy consumption in the summer and winter were as expected in this study, as shown in Figure 5a,b. Electric vehicles are assumed to be charged only at home, and the appropriate charging level for the EV in the home is between 1.5 and 5 kW. The daily travel distance of an electric car is assumed to be 60 miles, which is the average distance of a trip in the Saud Arabia. Additionally, it is assumed that the electric car leaves the house at 8:00 a.m., returns at noon, departs again at 2:00 p.m., and returns at 5:00 p.m., as illustrated in (Figure 5i).

A behavioral system was analyzed, and its ability to respond reliably to diverse energy needs was determined by measuring the balance between production and demand. In fact, Figure 5e,f show the energy balance achieved between demand and production between winter and summer, allowing the system condition to be examined and controlled (excess/deficit). The excess and deficit transition phases are depicted in Figure 5g,h during the operation of the system in winter and summer, respectively. Thus, the EMS’s supervision of the transition between states determines the energy distribution flows and the behavior of the system.

The home energy price signal is illustrated in Figure 5j, which depicts time-of-use (TOU) charges for resident users. This figure demonstrates the energy pricing pattern with term TOU at different periods during the day, including off-peak, average, and peak prices. The figure shows high prices during on-peak as compared with those in Figure 5e,f when the demand exceeded the generation. It incentivizes users to charge their stationery battery energy storage and EV at low electricity rates, which can then be used to meet load demands or resold to the grid at high electricity prices for economic gain.

### 4.1. Load Scheduling Capability Analysis

**Figure 6:**
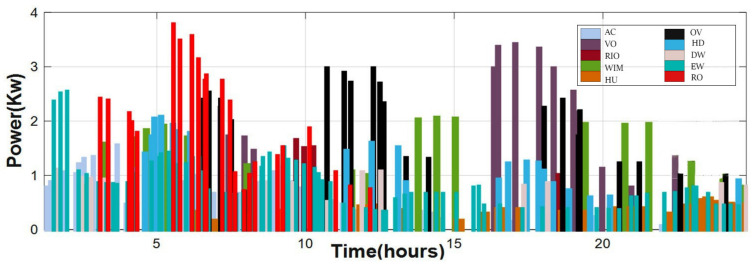
The optimal household load-scheduling pattern.

The load distributions of appliances (shiftable and non-shiftable) depending on time slots are shown in Table 2. During the first and second periods, the shiftable appliances used approximately 19.1 KW. While the total number of times ahead is equal to 24 times, the time intervals required for each device to function are shown in the same table. Figure 6 illustrates the appropriate home load scheduling profile. These households execute their given duties efficiently within the specified periods, and even non-shiftable homes operate continuously. Most homes run when the amount of power generated by SPV panels is high; 9 a.m.–3 p.m., according to Figure 5e, or the real-time energy price is low during off-peak periods, according to Figure 5j. Similarly, SPV power is employed to balance home power usage before it can be used to charge V2H. Table 2 shows the operating intervals for each device, with a total of 24 times ahead. Indeed, it summarizes the household attributes and total usage time according to the appropriate schedule.

### 4.2. Impact of V2H/GEV on Household Energy Consumption

**Figure 7:**
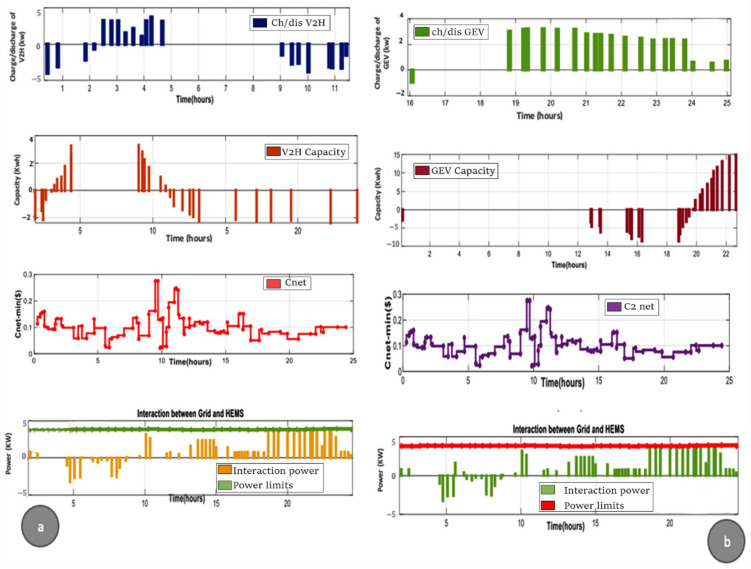
Amount of charging/discharging power (**a**,**b**); overall power storage capacity of V2H/V2G (**a**,**b**); amount of power traded between Grid and HEMS (**a**,**b**).

In the above scenarios, SPV energy is used to improve household energy consumption, and therefore, SPV energy is used to charge the V2H (see Figure 7). If SPV surplus remains, it is sold back to the grid, as shown in Figure 7a. Additionally, by 8 p.m., the power generated by the home SPV panels is minimum or non-existent, which requires charging the GEV through the grid (Figure 7b). Thus, the proposed HCPV system can be considered to be a financial benefit to domestic consumers and, at the same time, promotes the use of renewable energy during the periods of 8:35 a.m., 9:50 a.m., 5 p.m., 6:30 p.m., and 8 p.m. Indeed, it provides power data from the V2H to either meet the total power required or contributes during insufficient power. Since SPV production is sufficient to power the required homes, there is no need to purchase additional electricity from the grid during other high-cost periods such as 12:35 a.m. and 3:32 p.m. Due to V2H’s limited capacity and SPV output depending on the intensity of sunlight, V2H cannot be charged until 7:40 a.m. the day before with no SPV production and no additional electricity. For example, at around 8 p.m., the GEV helps V2H discharge, thus, reducing the power consumed by the network during peak hours, as shown in Figure 7a,b. The GEV is charged only during low hourly cost periods (Figure 7b). Preparing the battery to its full charge before departure guarantees driver satisfaction. Its stored capacity and charging/discharging force are within the mentioned limitations. Specific V2H and GEV discharge cost limits reduce charge/discharge cycles, thereby, increasing battery life. The interaction between the grid and HEMS is presented (Figure 7a,b).

### 4.3. Transferring SPV by V2H during Four Scenarios

**Figure 8:**
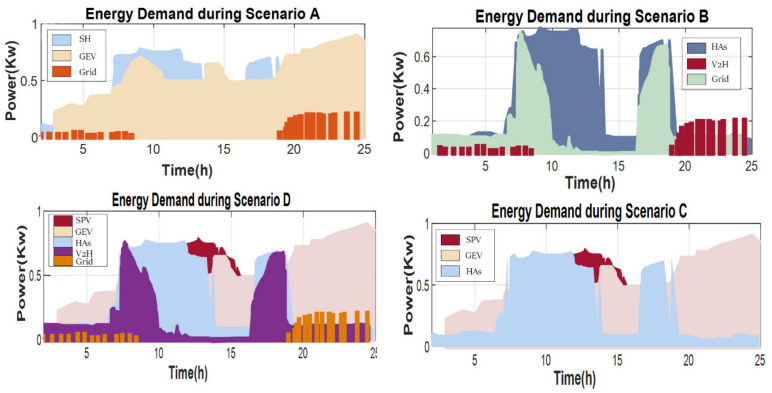
HCPV energy demand during four scenarios.

The V2H/BT is charged in Figure 8 to power the monitoring appliances. Before the micro-grid expands and retracts with GEV, the V2H/BT is discharged between 7:30 a.m. and 8:00 a.m. The V2H/BT is charged to power the supervisory devices, as depicted. Before GEV was identified and attached to the micro-grid, V2H/BT was discharged between 7:30 a.m. and 8:00 a.m. Scenario A shows that GEV and microgrid energy exchange was not required during this period, hence, relays ψ2 and ψ4 remained available. However, as solar energy exceeded demand, the SOC_V2H_ began to recharge, as shown in Scenario B. This value dropped again around 5:00 p.m. The charge lowers swiftly to 600 W at 1:30 p.m., allowing the SOC_GEV_ to improve to 68%. Extending GEV charging hours at night or during peak periods increases grid power demand when the GEV battery powers the residence. In Scenario C, the GEV may deposit extra SPV power in the V2H battery and use it when solar is available to support the home during power outages. Scenario D shows how V2H and SPV impact on household electricity use can substantially lower peak demand. This is twice the price of residential electricity in Saudi Arabia. V2H’s highest energy delivery will increase demand while saving money.

### 4.4. Impact of Travel Distance on V2H

**Figure 9:**
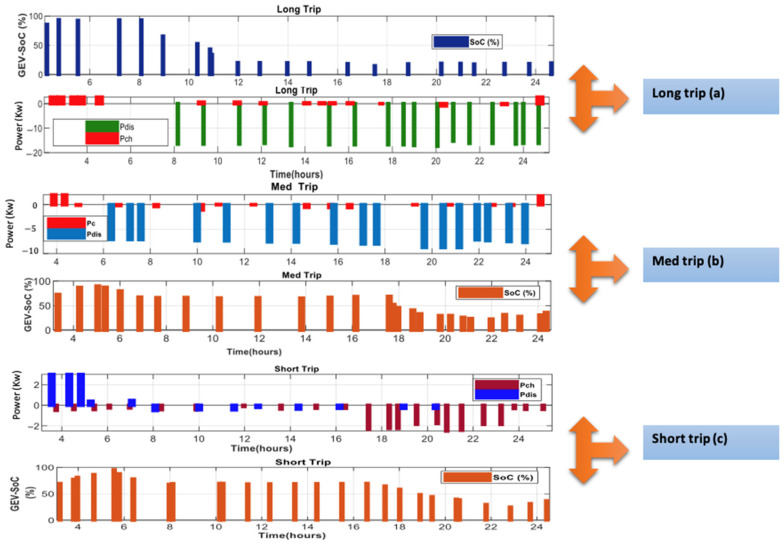
Influence of the travel distance on SoC_V2H_.

In Saudi Arabia, scheduling day trips are divided into three categories: (1) average travel distances of 80, 120, and 180 km are calculated for ease of calculation. Vehicle motion activity is vital to perfect vehicle control as this varies greatly. Due to the expansion of the public transport system in Saudi Arabia, the population is highly dependent on such transportation, however, private car trips are simple. Therefore, vehicle travel characteristics vary on weekends and business days. Vehicle travel affects SoC_V2H_ for short, medium, and long trips (Figure 9):

- Short travel distance, the SOC_V2H_ starts dropping less than 50% from 7:30 p.m.;
- Medium travel distance, the SOC_V2H_ starts dropping less than 50% from 6 p.m.;
- Long travel distance, the SOC_V2H_ starts dropping less than 50% from 10:30 a.m.

Generally, full-day load power distribution and excess solar energy distribution vary for different climatic conditions: sunny or cloudy. The maximum demand for household energy is between 5 p.m. and 10 p.m.

### 4.5. Analysis of Energy Flows and Cost Expenditure

#### 4.5.1. State 1: Standard GEV Charging

**Figure 10:**
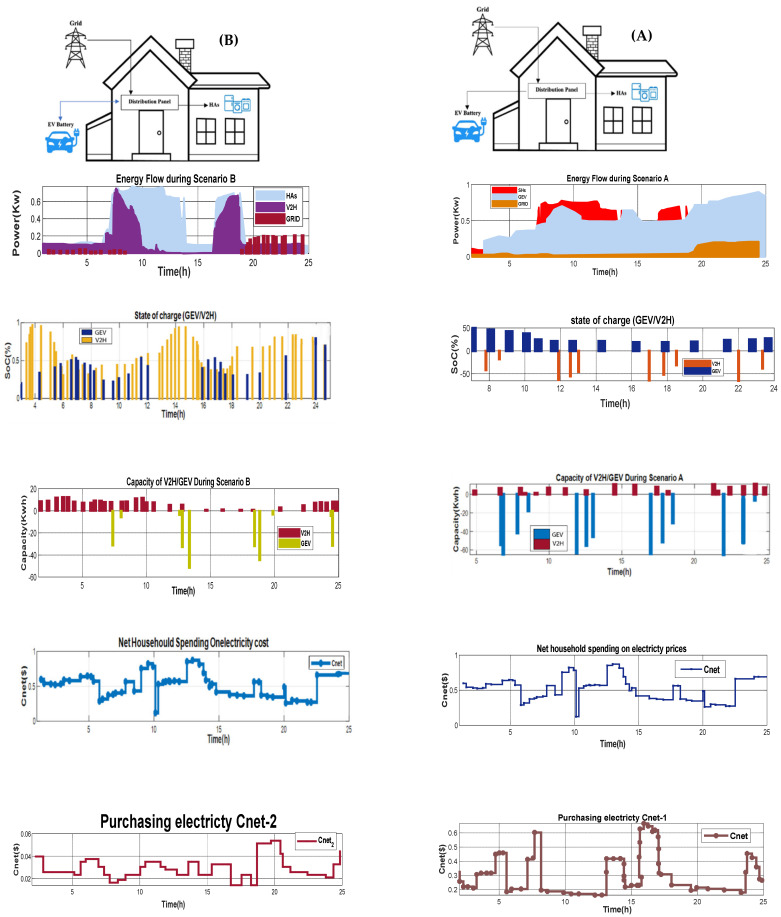

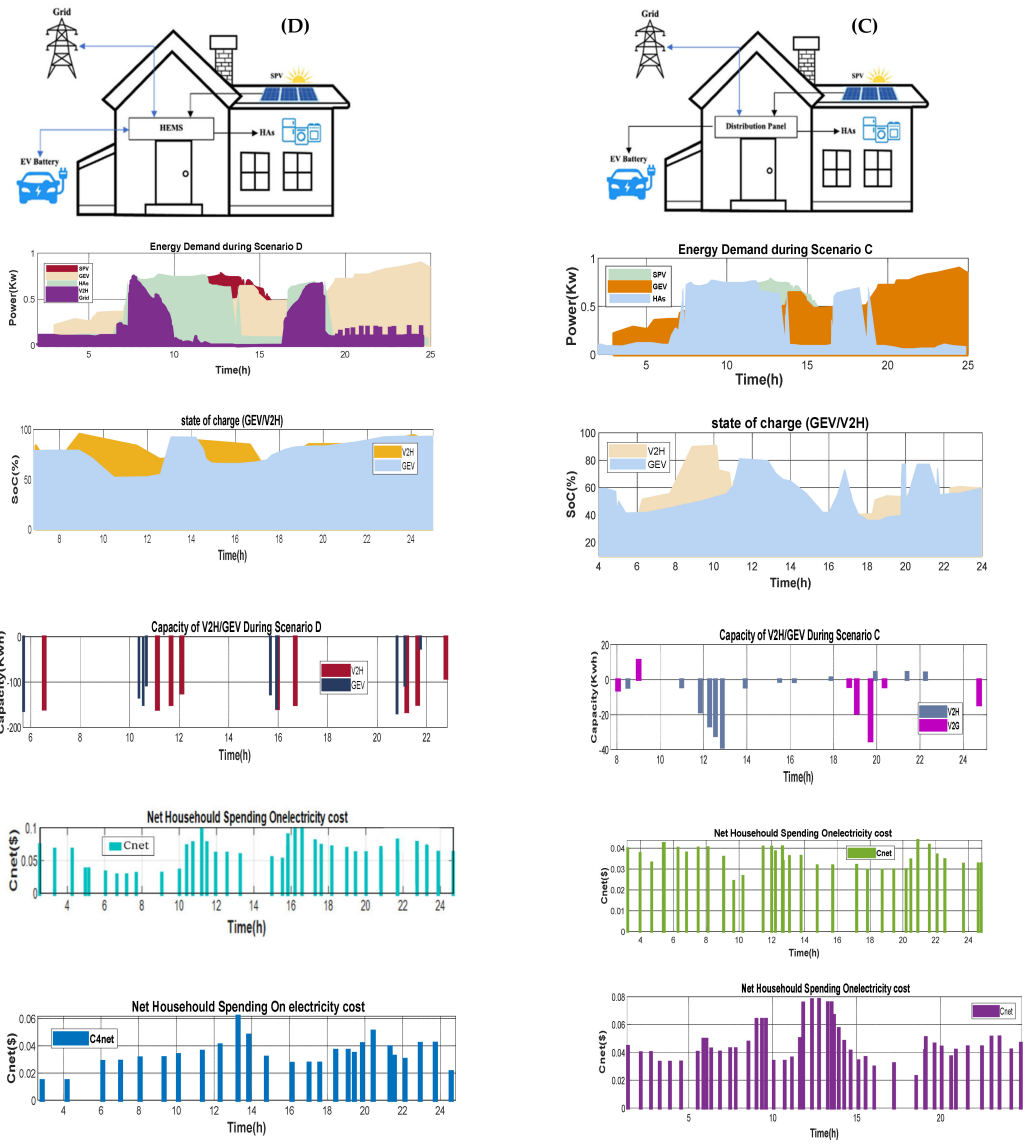
Energy flows and cost expenditure comparison for the four scenarios (**A**) Energy flows and cost expenditure of Scenario A (**B**) Energy flows and cost expenditure of Scenario B (**C**) Energy flows and cost expenditure of Scenario C (**D**) Energy flows and cost expenditure of Scenario D.

**Figure A2:**
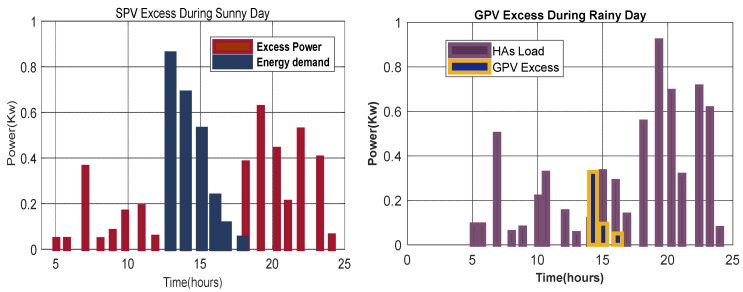
Hourly surplus GPV power during sunny and rainy periods.

The GEV is discharged with the grid from 7:30 to 8:00 a.m. No power exchange is required either for the vehicle or the grid. The HAs load subsequently increased by 700 W. The GEV fulfilled the lowest demand for energy. The solar energy peaked around 10:00 a.m. When solar energy exceeded the demand for HAs load, SOC_GEV_ began charging. The result of this mode is shown in Figure 10a. Due to limitations of the bi-directional charger, the charging schedule was optimized. Surplus energy was saved, and the demand for the GEV increased by 3 kW (see Figure A2). The household battery has a maximum charging capacity of 3 kW. The fee is terminated when the SOC is 10%. The model shows batteries charging when SPV is available; the power price is low at night, while peak demand occurs during charging, as shown in Figure 10a. Peak demand is limited to the maximum charging rate of each system.

#### 4.5.2. State 2: Standard V2H Charging/Discharging

The BT/V2H and V2G can be charged from the grid at night. This reduced the energy required to assess the vehicle. The GEV and grid became available to set the V2H due to when the GEV was fully charged. As stated before, the higher profit potential more than offsets the increased power usage. SoC_GEV_ and SoC_V2H_ remained at 28% and 82%, respectively; thus, the GEV was nearly empty (P_c,max_ = P_GEV,min_). Figure 10b shows the peaking requirements of up to 3 kW (the V2H’s maximum charge limit) and 2.8 kW, and 2.9 kW (the V2H’s maximum charge limit). When applied on the V2H, this tariff’s SOC stays above 40% for approximately 24 h. In the GEV, SOC dips below 40% for nearly a day.

#### 4.5.3. State 3: GEV Charging Supplemented by SPV

The SPV in this scenario interacted with the GEV and GEV-supported HAs, replacing only grid power, C_net_, with a minimum of $C_{\mathrm{net}}^{3}$. Equation (13) estimated the total number of C_HAs_ and SPEs ($C_{V2H}^{3}$). SPV may power the house in this case, while the GEV is mostly loaded (V2H = 0). Figure 10c shows the SPV smart home system and grid power supply system (see Figure 10c). The SPV/V2H may be charged from the network at night. This significantly reduced the amount of energy required to charge the car at home. Since the GEV was sometimes fully charged, more solar energy was available to charge the GEV/V2H (P_c,max_ = P_max GEV_). Microgrid usage rises, but potential profits offset this. SoC_GEV_ and SoC_V2H_ remained at 28% and 80%, respectively, with the GEV almost completely depleted in Figure 10c because of this situation. The bulk tariff in Figure 10c shows requirements of up to 6 kW (maximum charging) and peak requirements of up to 5 kW in the middle of the afternoon and at night, when electricity prices drop almost every day because of dynamic changes in electricity prices. Due to higher SOC levels on most days, charging at night is less frequent with this tariff than with the previous two. In contrast to time-of-day prices, the dynamic adjustment in energy currency values at the slightest rise in demand peaks throughout most of the week.

#### 4.5.4. State 4: V2H Charging/Discharging Supplemented by SPV

SPVs interact with HA groups in this scenario, SPVs interact with V2H groups, and V2H/GEV communicate with the HA groups. In terms of C_net,min_ = $C_{\mathrm{net}}^{4}$, Equation (14) contains the projected total expenditures for all health and social service entities (HSSEs) ($C_{\mathrm{HAs},\mathrm{SPE}}^{4}$). In addition, C_HCPV-V2H_ is equivalent to $C_{\mathrm{HCPV}-V2H}^{4}$. By 7:00 p.m., this project will be fully powered by renewable energy. V2G options are available from 8:00 a.m. to 8:00 p.m., while V2H is available all night. Because of V2G, energy savings of 40% were achieved. However, it is necessary to evaluate the amount of energy used in the house. V2G is an efficient way to save energy when the GEV and SPV are insufficient to meet demand. Although the number of cars lost due to SPV (P_min-ch_) charging and discharging is significant, the cost-effectiveness of this solution depends on whether energy rates are low at night or during the day. When refilling the V2H, an electric car driver may return to base overnight (P_max-ch_). SOC_V2H_ had a 46% success rate when the SPV was low. The GEV is completely discharged from about 7:00 a.m. The V2H will be charged until 10:00 a.m. The GEV charging will start at 10:00 a.m. and will be activated at 7:00 p.m. The result of this situation is shown in Figure 10d.

To sum up, the four scenarios have been compared in terms of energy demand, SoC and capacity of V2H and the GEV, net household spending electricity cost, and net purchasing electricity cost. The bi-directional power converter used for the V2H unit in Scenarios B and D minimizes the energy required to assess the GEV as compared with Scenarios A and C. However, Scenario D depends on RnEs which significantly impact the SOC_GEV_ and SOC_V2H_. Scenario C performs better than Scenarios A and B in term of frequent charging at night due to sufficient SOC levels from SPV power generation. Scenarios A and B are highly affected by the TOU tariffs, whereas Scenarios C and D are affected by whether energy rates are low at night or during the day.

### 4.6. Daily Household Electricity Expenditure

**Figure 11:**
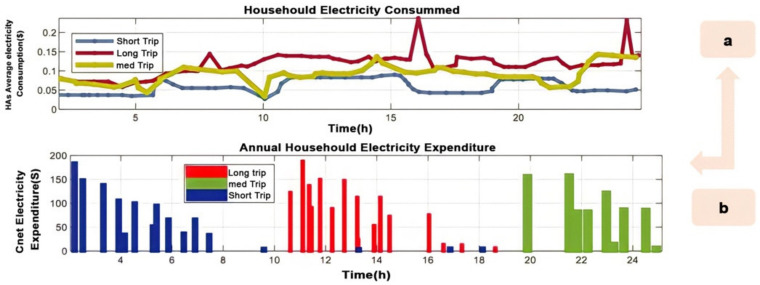
(**a**) Household annual average cost during long, medium, and short trips; (**b**) annual HCPV electricity expenditure.

Figure 11 illustrates how much household power is consumed in each trip, the average home energy C_net_, and the minimum cost in each trip. Additionally, C_net_ increases in value because of the GEV trip. The typical annual residential energy cost (C_net,min_) is shown in Figure 11a,b. Minor- and travel-related minimums decrease by 28%, 23%, and 19%, respectively, for V2H without SPV and by 24%, 19.9%, and 9.97%, respectively. Both Scenarios A and B are recommended for medium- and long-distance travel. When both are operated from a single power source, the SPV mode has a lower cost of spending than the V2H mode. Grid power and SPV sales cost are different, with the latter having higher point pricing. Annual net expenditures with SPV decreased by 38%, 30.9%, and 31.5%, respectively, due to SPV’s superior cost performance. During short trips, travel lowers C_net_ by 51, 42, and 40.2%, respectively. On the other hand, medium trips reduce the annual C_net_ by 50.5%, 41.5%, and 30%, respectively. The yearly C_net_ in SPV-V2H mode decreased by 48.5%, 46.85%, and 31%, respectively, while travelling long distances. Along with the economic advantages of utilizing V2H with SPV for different modes of transport, the data below demonstrate the technology’s potential. Additionally, weather impacts HCPV income and sustainable energy use.

However, energy sustainability using V2H technology has its limitation as the SG depends on the GEV existence and being plugged in. Thus, including prediction of the GEV driving patterns and DR is vital to balance the demand and response. Furthermore, uncertainty of solar power generation, load, market prices and user behavior should be considered during scheduling to optimize energy management.

## 5. Conclusions

This study employs V2H technology to discuss the impact of renewable energy systems, which can better handle peak energy demand while simultaneously reducing prices. The HCPV solar energy and energy recovery systems were set up with this in mind. The backup energy recovery device recovers deficit power (V2H). A power shortage is alleviated thanks to V2H energy harvesting during peak hours. Additionally, our work designed and implemented an accurate HEMS strategy. The proposed approach aims to regulate energy production and consumption. Thus, the HCPV system is based on the two operating modes: storage and recovery. There is no doubt that the proposed approach can intelligently manage the proposed system to prevent unexpected fluctuations. The effects of V2H and/or HCPV on residential energy consumption and net electricity consumption are explored and discussed. In addition to driving hours, the weather is also considered for the GEV. The obtained results demonstrate that the HCPV system provides acceptable quality charging power with resistance to unwanted fluctuations, as recommended by the HEMS. Our proposed algorithm shows that energy savings up to 40% were achieved when V2H is integrated with an intelligent HEMS. Furthermore, the MAS simulation indicates that the state of charge of V2H had a 46% success rate when the solar power generation was low.

Future research should focus on embedded systems due to delays in these systems. Real-time operations save money and energy while improving power design based on variations in how electricity is metered.

## Figures and Tables

**Table 1:** Agents and their roles.

Agent Name		Agent Role
Sink	Primary agent	Coordinates with intermediate-level agents
With intermediate-level agents	Renewable energy agent	Collects real-time energy demand data to assess whether there is an energy surplus or deficit
	EV agent	Charging and discharging GEV batteries
	V2H agent	Regulates the output power to provide backup power during power shortage conditions
	Stationary battery agent (SBA)	Storing excess energy and activated when a power deficit occurs

**Table 2:** Household parameters (non-shiftable appliances are highlighted with gray color) [39].

Households	Starting Time	Ending Time	Total Running Duration	Minimal Power (kW)	Maximal Power (kW)
Washing machine (WM)	9 pm	10 pm	1 h wash	1.50	2.5
Vacuum cleaner (VC)	1 pm	3 pm	0.50 h	1.25	2.1
Air conditioner (AC)	8 am	8 am		3.0	4.1
Rice cooker (RIC)	10 am	12 pm	0.75 h	0.80	1.1
Humidifier (HU)	8 am	12 am	4 h	0.160	0.50
Computer phones light (CPL)	8 am	8 am	Full time-24 h	1.20	1.7
Oven (OV)	5 pm	7 pm	2 h	2.0	5.0
Hairdryer (HD)	8 am	8.30 am	30 min	1.80	1.90
Dishwasher (DW)	8 am	11 am	3 h	1.8	2.4
Electrical water (EW)	8 am	8 am	Full time-24 h	1.5	2.3
Robot (RO)	11 am	12 pm	2 h	2.0	3.6

## Data Availability

Not applicable.

## Nomenclature

**Acronyms**	
HCPV	Home centralized PV
MAS	Multi-agent system
IA	Intelligent agent
GEV	Green electric vehicle
SPV	Solar photovoltaic
HEMS	Home energy management
HAs	Home appliances
SH	Smart home
SB	Stationary battery
MG	Microgrid
SG	Smart grid
V2H	Vehicle-to-home
RnEs	Renewable energies
Ah	amp hours
**Sets**	
P_HCPV,I_	Grid power requirement
g_Home_,_i_	Home electricity demand
P_SPVt,s_	SPV supply
ψ_1,2,3_	Relay states 0/1
I	Time index
δ	Discharge depth
S(i)	V2H/GEV state in/out
SOC	State of charge
T	Maximum discharge
PC_max_	Max power for charging
P_Bch/disch-_	max: Max battery
Ts (s)	Starting Time Interval
C_net-min_	Household elec. costs
P_HCPV,I_	Grid power requirement
**Parameters**	
P_PV,comp_	SPV consumption rate
P_SPV,T_	Total SPV energy all/days
δ_SPV_	Investment cost ($/Kw)
P_Bch_/_disch,i_	Battery capacity, KW
SPV_t,s_	Solar power supply
PC,i	Maximum amount of energy
P_ST_	Saved power during time t
g_GEV,i_	V2H-PEV battery charger
ηch/ηdisch	Charging/discharging (%)
χ	Daily driving distance (KM)
*Dβ*	GEV max-distance (KM)
C_HAs-SPE_	Total sp on purchasing
C_HCPV-SPV_	Cost of installation
C_HCPV-TR_	Forecasts total revenue
P_SPVt,s_	Solar power at time t
G_SPVt,s_	Solar irradiance
**Average Temperature**	
P_SPV,t_	SPV irradiance at time t
P^rated^	Output power rated (KW)
G_GEV,i,max_	Max V2H-PEV charger
∆c	Charge
∆d	Discharge

## Appendix B

**Table A1:** Input parameters of HCPV system.

Items	Parameters
HCPV System Technical Parameters	
SPV related power	1.0 Kw
Interest rate	4.80%
HCPV lifetime	25.0
Rated Capacity: g^rated^_SPV (KW)_	8.02 Kw
Investment cost (δ_SPV_) ($/KW)	769.0 $/KW
SPV Cell Numbers	Ns 3; Np 6
P_Grid,max_ (Kw)	9725 Kw
**GEV Technical Specifications**	
V2H Energy Efficiency (KWh/100 Km)	10.0 KWh/100 Km
P^chmax^_GEV_ (KW) = 5.0	C^Max^_GEV_ (KW) = 30
P^Dismax^_V2H_ (KW) = 0.95	C^Min^_GEV_ (KW) = 5.0
η^ch^_GEV_/η^disch^_GEV_=	
C^dis^net (RTP_GEV_) = 1.85 RTP_Avg_	
Daily Driving Distance (Km)	32.0 Km
Maximum Charging Power (P_Bdich_max) (Kw)	3.0 Kw
SoC_V2H-min_ (%)	19%
SoC_V2H-max (_%)	90%
SOC_GEV_ Range	30~80%
**GEV Technical Specifications**	
Weather Data	From 2014 to 2019
C^Max^_V2H_ (KW) = 8.0	η^ch^_V2H_/η^disch^_V2H_=
C^Min^_V2H_ (KW) = 3.0	C^dis^net(RTP_V2H_) = 1.2 RTP_Avg_
P^chmax^_V2H_ (KW) = −3.5	C^Max^_V2H_ (KW) = 8.0
P^Dismax^_V2H_ (KW) = 0.95	
**Probability Distribution of Daily Travel Behavior**	
GEV parked Home (H)	From 7 p.m. to 7 a.m.
Short Distances Km	8~30 km
medium distances Km	50~80 km
Long Distance Km	100~120 km
